# Multi-stage glaucoma classification using pre-trained convolutional neural networks and voting-based classifier fusion

**DOI:** 10.3389/fphys.2023.1175881

**Published:** 2023-06-13

**Authors:** Vijaya Kumar Velpula, Lakhan Dev Sharma

**Affiliations:** School of Electronics Engineering, VIT-AP University, Amaravati, Andhra Pradesh, India

**Keywords:** convolutional neural network, classifier fusion, deep learning, fundus image, hybrid model, transfer learning

## Abstract

**Aim:** To design an automated glaucoma detection system for early detection of glaucoma using fundus images.

**Background:** Glaucoma is a serious eye problem that can cause vision loss and even permanent blindness. Early detection and prevention are crucial for effective treatment. Traditional diagnostic approaches are time consuming, manual, and often inaccurate, thus making automated glaucoma diagnosis necessary.

**Objective:** To propose an automated glaucoma stage classification model using pre-trained deep convolutional neural network (CNN) models and classifier fusion.

**Methods:** The proposed model utilized five pre-trained CNN models: ResNet50, AlexNet, VGG19, DenseNet-201, and Inception-ResNet-v2. The model was tested using four public datasets: ACRIMA, RIM-ONE, Harvard Dataverse (HVD), and Drishti. Classifier fusion was created to merge the decisions of all CNN models using the maximum voting-based approach.

**Results:** The proposed model achieved an area under the curve of 1 and an accuracy of 99.57% for the ACRIMA dataset. The HVD dataset had an area under the curve of 0.97 and an accuracy of 85.43%. The accuracy rates for Drishti and RIM-ONE were 90.55 and 94.95%, respectively. The experimental results showed that the proposed model performed better than the state-of-the-art methods in classifying glaucoma in its early stages. Understanding the model output includes both attribution-based methods such as activations and gradient class activation map and perturbation-based methods such as locally interpretable model-agnostic explanations and occlusion sensitivity, which generate heatmaps of various sections of an image for model prediction.

**Conclusion:** The proposed automated glaucoma stage classification model using pre-trained CNN models and classifier fusion is an effective method for the early detection of glaucoma. The results indicate high accuracy rates and superior performance compared to the existing methods.

## 1 Introduction

Glaucoma can gradually impair the optic nerves, which may lead to blindness and permanent vision loss. The damage to the optic nerve is commonly caused by an increase in the intraocular pressure (IOP), which is the fluid pressure in the eye (Sommer et al., 1991). For those over the age of 60, glaucoma is one of the major causes of blindness. It can occur at any age, but is more common in elders. Glaucoma is estimated to affect 80 million people in 2020, with that number rising to 111 million by 2040 (Tham et al., 2014). The majority of glaucoma-related visual loss can be averted with early detection and treatment. As a result, detecting glaucoma at an early stage is essential. Manual glaucoma screening for all suspects is difficult and time consuming due to the shortage of experienced ophthalmologists. It is necessary to develop an automated glaucoma diagnosis system that is both accurate and efficient. There are different methods for the detection and classification of glaucoma using fundus images (Shabbir et al., 2021). One way to detect glaucoma is to look for structural changes in the eye. Ophthalmologists examine the inner features of the eye with fundoscopy and optical coherence tomography (OCT) (Chan et al., 2019) to diagnose abnormalities (Sułot et al., 2021). In the other way, the ratio of the size of the optic cup (OC) to the size of the optic disc (OD) and the structure of these two regions are crucial markers in fundus imaging used to diagnose glaucoma.

The machine learning methods such as artificial neural networks (ANNs), support vector machine (SVM) (Roy et al., 2016; Raghavendra et al., 2018), k-nearest neighbor (k-NN) (Sharma and Sunkaria, 2018; Sharma et al., 2021), and random forest (RF) are other ways for detection and classification in medical imaging problems. The OC and OD can be examined with machine learning algorithms (Mookiah et al., 2012; De La Fuente-Arriaga et al., 2014; Kausu et al., 2018; Soltani et al., 2018; Tulsani et al., 2021). As a result, prior studies suggested calculating glaucoma-related characteristics such the cup-to-disc ratio (CDR) (Haleem et al., 2018), the inferior superior nasal temporal (ISNT) rule, the glaucoma risk index (GRI), and the neuroretinal rim (NRR). All of these include manually labeling the cup and disc in each image. As a result, it takes time and is labor intensive. Many image processing tasks, such as classification and diagnosis, can be carried out with deep learning models (Anwar et al., 2018; Islam et al., 2018; Shorten et al., 2021). Such models can detect various attributes in incoming images without the use of pre-processing techniques such as segmentation (Cheng et al., 2013; Krishnan and Faust, 2013; Mohamed et al., 2019; Zulfira et al., 2021) and texture feature-based approaches (Bock et al., 2007; Acharya et al., 2011; Haleem et al., 2016). Deep learning approaches (Christopher et al., 2018; Serener and Serte, 2019; Sreng et al., 2020) are the better option for image classification problems. They perform admirably in a variety of fields, particularly image classification. These algorithms have significantly improved the performance of fine-grained classification tasks that aim to differentiate between subclasses.

The motivation of this paper is to address the critical issue of glaucoma, an eye problem that can lead to vision loss and even permanent blindness, if left undiagnosed and untreated. Traditional glaucoma diagnosis techniques are frequently labor intensive, inaccurate, and time consuming, which can postpone the disease’s discovery and have more serious consequences. Hence, there is a need for automated glaucoma diagnosis with high accuracy to enable early detection and prevention of the disease. To address this need, the authors proposed a model for automated glaucoma stage classification using pre-trained deep convolutional neural network (CNN) models (ResNet50, AlexNet, VGG19, Dns201, and IncRes). The model is tested on four public datasets, and a classifier fusion technique is employed to improve the overall system performance.

The main contributions of this paper are as follows: (i) Three-class glaucoma classification. (ii) k-fold cross-validation is used for each model, and performance metrics are discussed. (iii) For performance improvement, classifier fusion is used. (iv) Visualization techniques are used to determine which part of the image is used more to predict the class the most.

The rest of the paper is organized as follows. We discuss the state of the art on glaucoma detection and classification in Section 2, the collection and preprocessing of the dataset in Section 3, and basic CNN architecture and also the classifier fusion in Section 4. The results of all CNNs and classifier fusion are discussed and all output graphs and related tables are shown in Section 5. In Section 7, the conclusion of the proposed work is discussed.

## 2 Related work

Medical diagnostic systems, especially in current ophthalmology, are increasingly repleted with image processing techniques. The retinal images reveal information about the health of the visual system. A computerized system enables standardized wide-scale screening at a lesser cost, minimizes human mistakes, and can extend services to rural and isolated places when an ophthalmologist is not available, as well as when numerous patients need to be diagnosed. Over the last couple of decades, research has been carried out to create automated approaches. Cheng et al. (2013) presented the approach of superpixel classification for glaucoma detection in the optic cup and optic cup segmentation. In optical disc division, histograms and focus encompass insights are used to classify every superpixel as a disc or non-disc. Hatanaka et al. (2009) suggested a technique for computing the CDR using a vertical profile on the optic disc. By evaluating the second-order differential values of the vertical profile on the disc area, the boundary between the cup and disc regions was discovered. The CDR was calculated at that moment, and that was used to determine if the patient had glaucoma. Parashar and Agrawal (2020a) also proposed a novel approach for the automatic classification of glaucoma stage categorization based on 2D tensor empirical wavelet transform (2D-T-EWT). Parashar and Agrawal (2020b) proposed a novel approach for glaucoma stage categorization based on the flexible analytic wavelet transform (FAWT).

A large portion of the proposed approaches for automated glaucoma recognition used underlying elements (CDR, RDR, and ISNT) (Cheng et al., 2013; Mohamed et al., 2019) or intensity-based features independently. Glaucoma classification, based on structural variations, can sometimes produce incorrect results due to erroneous cup or disc segmentation due to the presence of light lesions. Using a combination of texture and higher-order spectra (HOS) data from digital fundus pictures, Acharya et al. (2011) proposed a unique approach for glaucoma identification. Support vector machines, sequential minimal optimization, naive Bayesian, and random-forest classifiers are utilized for supervised classification. Diaz-Pinto et al. (2019a) proposed a new retinal image synthesizer and a semi-supervised learning approach for glaucoma assessment based on deep convolutional generative adversarial networks (GANs). This system can not only create images synthetically but it can also dynamically supply labels. Sreng et al. (2020) described an automated two-stage glaucoma assessment method that reduces ophthalmologist’s work. Using a DeepLabv3+ architecture, the device first segmented the optic disc region but replaced the encoder module with multiple deep CNNs. Olivas et al. (2021) proposed a transfer learning technique for glaucoma detection using the MobileNet and Inception V3 models with images of the retina. Li et al. (2019) offered an attention-based CNN (AG-CNN) for glaucoma detection that addresses the drawback that existing techniques can easily reduce excessive redundancy in fundus pictures for glaucoma identification, possibly lowering glaucoma detection reliability and accuracy. Imran et al. (2021) proposed a new hybrid convolutional and recurrent neural network (CRNN) for cataract classification based on fundus images. They have used AlexNet, GoogLeNet, ResNet, and VGGNet to extract multilevel feature representation and assess how effectively these model works in cataract classification when combined with transfer learning. G´omez-Valverde et al. (2019), in their proposed study, have used several CNN schemes to demonstrate the impact of significant aspects such as dataset size, architecture, and the usage of transfer learning *vs.* newly created architectures on performance. Diaz-Pinto et al. (2019b) used five ImageNet-trained models for autonomous glaucoma assessment utilizing fundus images in this research (VGG16, VGG19, Inception V3, ResNet50, and Xception). The results of a thorough validation employing cross-validation and cross-testing methodologies were compared to those of previous studies. Sułot et al. (2021) designed a novel task-specific CNN architecture for glaucoma image categorization. Five machine learning algorithms for classifying glaucoma based on retinal nerve fiber layer (RNFL) thickness—a well-known biomarker in glaucoma diagnostics—an ensemble classifier based on Inception v3 architecture, and classifiers based on image attributes were all investigated.

Deep learning algorithms have increased the state of the art in medical image classification and also ophthalmic image classification, segmentation (Sreng et al., 2020), and detection of diseases over the last few years. In ophthalmology, deep CNNs (Liao et al., 2019; Serte and Serener, 2019) were employed for a variety of tasks, such are detecting glaucoma and analyzing and segmenting optical coherence tomography (OCT) images. Transfer learning has become an important strategy for applying features acquired to execute one task to other tasks in order to lessen these needs.

## 3 Collection of databases

In this section, dataset collection and preprocessing steps are used for fine-tuning the fundus image for further processing. The data preprocessing is essential for the transfer learning approach because each pre-trained deep CNN is trained for a specific input image size. In the image pre-processing phase, we have carried out image resizing and data augmentation, which are important techniques used in image classification, especially when working with pre-trained CNN models. These are typically trained on images of a specific size. If the images to be classified are of different sizes, they must be resized to match the input size of the CNN model. Data augmentation helps to increase the size and diversity of the training data. Both techniques help to optimize the performance of the pre-trained CNN model and increase its accuracy. The suggested model is trained and tested on both two-class datasets (ACRIMA and RIM-ONE) and three-class datasets (Harvard and Drishti), and the details of the datasets are shown in Table 1.

**TABLE 1:** Datasets used in this work.

Two-class datasets					
Dataset name	Image format	Resolution	Normal	Glaucoma	
				Affected	
ACRIMA; Diaz-Pinto et al. (2019b)	JPG	2048 × 1536	309		396
RIM-ONE; Fumero et al. (2011)	JPG	2144 × 2144	255		200

Three-class datasets					
Dataset name	Image format	Resolution	Normal	Early	Advanced
Harvard	PNG	240 × 240	788	289	467
dataverse (HVD); Kim. (2018)					
Drishti; Sivaswamy et al. (2014)	PNG	2048 × 2048	29	15	83

The ACRIMA database has 705 fundus images altogether, including 396 glaucomatous and 309 normal images. As part of the ACRIMA experiment, they were collected with the agreement of glaucomatous and healthy patients (Diaz-Pinto et al., 2019b). The eyes were dilated and centered in the optic disc when the majority of the fundus photos in this collection were taken. All the images from the ACRIMA database were annotated by two glaucoma specialists with 8 years of experience. No additional clinical information was taken into account while labeling the images. Another popularly used, freely accessible dataset is the RIM-ONE dataset for glaucoma classification and glaucoma detection (Fumero et al., 2011). To create the database, three Spanish hospitals Hospital Universitario de Canarias, Hospital Clinico San Carlos, and Hospital Universitario Miguel Servet worked together. There are 200 glaucoma images, 255 normal photos, and 455 fundus images in total. The Harvard dataset was originally collected from Kim’s Eye Hospital (Kim, 2018). The dataset was uploaded by Ungsoo Kim. There are 788 normal images, 289 early glaucoma images, and 467 advanced glaucoma images in the dataset. The dataset has already been preprocessed and is ready for deep learning tasks. It is available on the Harvard Dataverse and can be downloaded at Kim, Ungsoo, 2018, “Machine learn for glaucoma,” and Harvard Dataverse V1. The Drishti dataset was originally collected at Aravind Eye Hospital, Madurai, and consists of a total of 101 images (Sivaswamy et al., 2014). There are 50 training images and 51 test images. Clinical investigators chose their glaucoma patients based on their examination’s clinical findings. Male and female patients that were chosen for treatment ranged in age from 40 to 80. Each image was evaluated and classified as normal or glaucoma by a team of four glaucoma experts with varying levels of clinical experience. This dataset is available in two classes as normal and glaucoma. However, with reference to the work of Raja et al. (2021), we have modified it as the three-class dataset. Their research paper utilizes the white pixel-containing separated optic disc and cup region to determine the area and calculate the CDR value. Based on these values, three stages of glaucoma are classified. The CDR values are used to determine different stages of glaucoma. A CDR value less than 0.41 indicates normalcy, whereas a value greater than 0.41 indicates abnormality. An early-stage prediction is made if the CDR value falls between 0.41 and 0.5, whereas an advanced-stage prediction is made if the CDR value is greater than 0.5.

**FIGURE 1:**
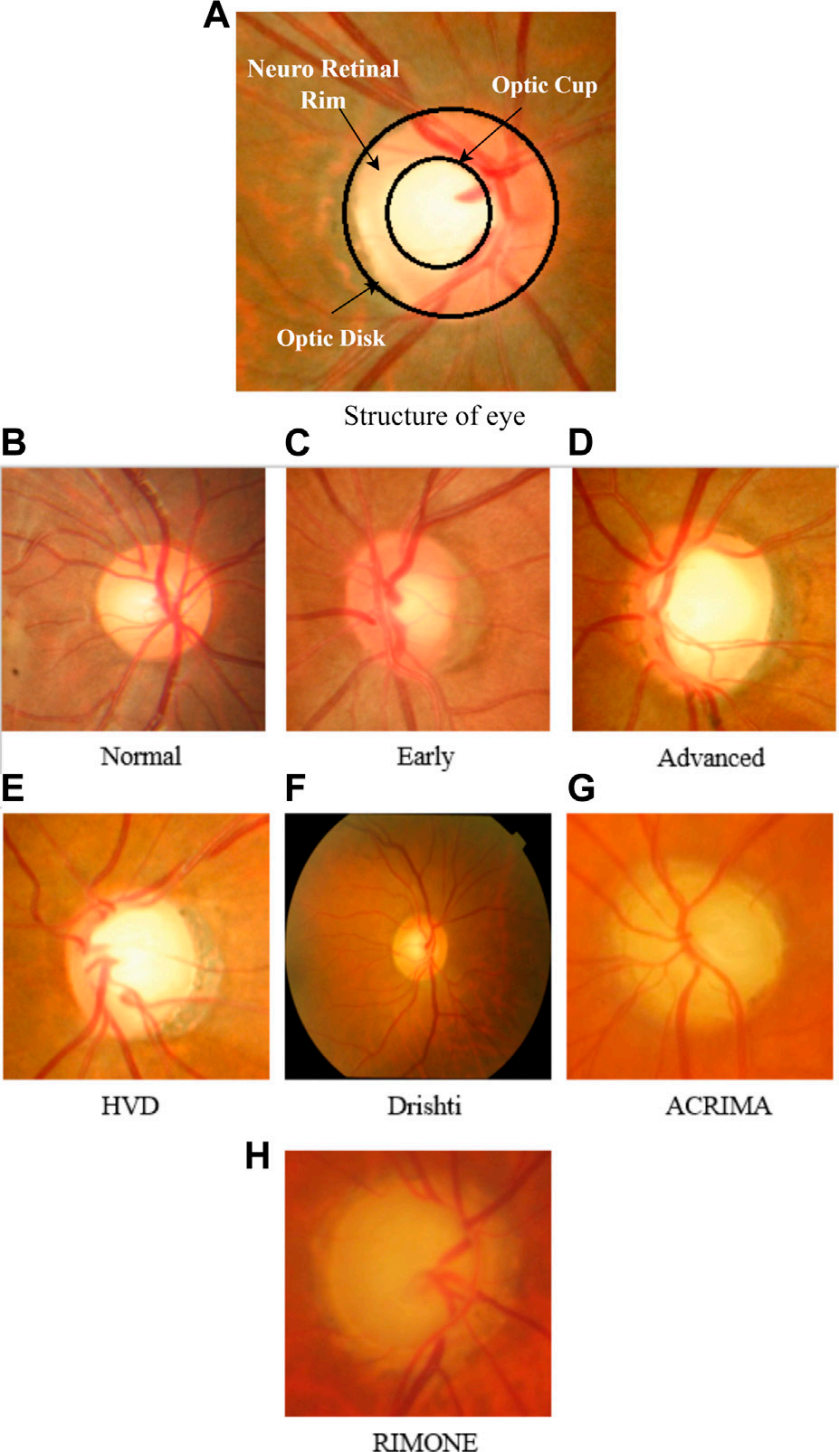
**(A)** Structure of the eye representing CDR and NRR. **(B–D)** Sample images of the HVD dataset to show the variation in the cup and disc ratio. **(E–H)** Sample glaucoma-affected images of datasets used in our work.

Figure 1A depicts the structure of the eye, representing CDR and NRR. The total color intensity and textural information rise in glaucoma images as the size of the cup and disc grow. We can see the variation of cup and disc ratio changes in images of the HVD dataset, as shown in Figures 1B–D. Some sample images of datasets that are used in this work are shown in Figures 1E–H.

## 4 Proposed methodology

**FIGURE 2:**
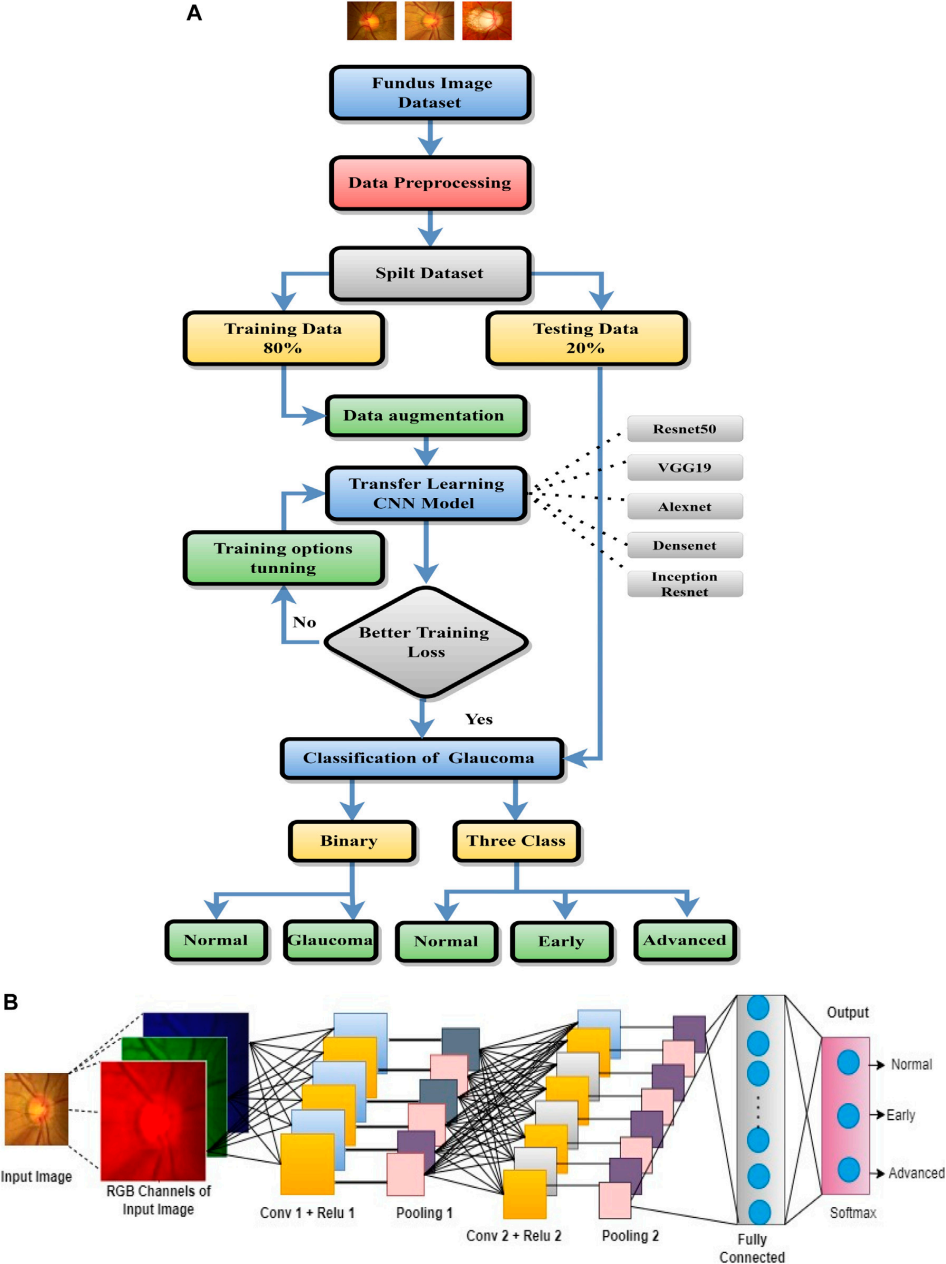
**(A)** Workflow diagram for glaucoma classification. **(B)** Basic CNN architecture.

The proposed system workflow is shown in Figure 2A, and it consists of the following steps: data collection and preprocessing, pre-trained CNNs selection for transfer learning, multi-stage classification, voting-based fusion, and performance evaluation. The aim of the transfer learning stage is to apply the knowledge of different pre-trained CNNs for glaucoma classification.

• Data collection and preprocessing: Four different datasets of retinal images are collected from various sources. The images are resized before being fed to the CNN to model since the CNN model is designed for specific input image sizes. Then, we applied data augmentation to increase the size and diversity of training data. The data collection and preprocessing steps were already discussed in Section 3.
• Pre-trained CNN selection: Different pre-trained CNNs such as ResNet50, AlexNet, VGG19, Dns201, and InsRes are selected. The selected pre-trained CNNs are used to extract high-level features from the retinal images.
• Multi-stage classification: Through transfer learning, the pre-trained CNNs are used for multi-stage classification. The images are classified as normal, early, or advanced glaucoma.
• Voting-based fusion: The results from multi-stage classification are fused using a voting-based approach. The final diagnosis is based on the majority of votes of different classifiers.
• Performance evaluation: The performance of the proposed methodology is evaluated using various metrics such as accuracy, sensitivity, specificity, and area under the curve (AUC). The results are compared with the state-of-the-art methods for glaucoma diagnosis.

### 4.1 Basic architecture of CNN

The basic architecture of CNN is shown in Figure 2B. The concept of simple and complex cells in the visual cortex in the brain- inspired the CNN, which is a basic type of deep learning approach (Liu et al., 2018; Alghamdi and Abdel-Mottaleb, 2021). Basically, the process of CNN will be performed through three layers: 1) convolutional, 2) pooling, and 3) fully connected (FC). The FC layers are trained for the final classification after multiple alternating convolutional and pooling layers. CNN has been frequently used in image classification tasks with great effectiveness.

The basic CNN operation for three layers deep can be expressed as a mathematical equation as follows:

$$f\;\left(x\right)=sign\;\left(2\sigma_{softmax}\;\left(W_{3}\sigma_{relu}\;\left(W_{2}\sigma_{relu}\;\left(W_{1}x+b_{1}\right)+b_{2}\right)+b_{3}\right)-1\right)$$ (1)

In Eq. 1, “*x*” is the input to the CNN which is to be given for the first convolutional (Conv) layer with rectified linear unit (Relu); then, this result is the input for second Conv layer with Relu and finally a third layer with softmax activation function, which is used for classification. Here, *W*_*l*1_, *W*_*l*2_, and *W*_*l*3_ are matrices which relate to hidden layers and *b*_1_, *b*_2_, and *b*_3_ are the bias functions.

#### 4.1.1 Convolutional layer

There are three operations in the Conv layer, namely, (i) convolution, (ii) batch normalization, and (iii) Relu.

(i) Convolution: The Convolution operation between input and filter weights is as follows:

$$y_{i}=w_{i}\;*\;x_{i}+b_{i}\;for\;i=1,2,\ldots ,N$$ (2)

where *y*_*i*_ is the output corresponding *i* ^*th*^ layer, *w*_*i*_ is the weight of *i* ^*th*^ layer, *x*_*i*_ is the input for *i* ^*th*^ layer, and *b*_*i*_ is the *i* ^*th*^ bais. N is the number of filters.

(ii) Batch normalization: During the training of deep neural networks, batch normalization is used to standardize the inputs to a layer for each mini-batch. This technique helps stabilize the learning process and reduces the number of training epochs required to create deep CNNs significantly. Typically, batch normalization is performed after the convolution operation to accelerate the training process.

$$y_{i}^{\prime }=\sigma \;*\;x{ˆ}_{i}+\beta$$ (3)

where *σ* and *β* are the scale and shift factors, respectively, and *x*ˆ_*i*_ is the normalized input.

(iii) Relu: It is defined as it gives “0” for all negative input values, and it gives “*x*” for all positive input values. This is the frequently used activation function in most applications.

$$y=\max\left(0,x\right)$$ (4)

where *y* is the output of relu function and *x* is the input.

#### 4.1.2 Pooling layer

To reduce the spatial size of the input, this layer is employed after a convolution layer. It is applied to each of the input volume’s depth slices separately. The pooling operation is performed by sliding a 2D kernel over each channel of the feature map, summarizing the features that fall inside the filter’s coverage zone (Rahul and Sharma, 2022). There are three types of pooling layers: max pooling, average pooling, and global pooling.

If the feature map has dimensions *n*_*h*_ *× n*_*w*_ *× n*_*c*_, then the output dimensions we get after the pooling layer are as follows:

$$\frac{\left(n_{h}-f+1\right)}{s}\times \frac{\left(n_{w}-f+1\right)}{s}\times n_{c}$$ (5)

where *n*_*h*_ is the height, *n*_*w*_ is the width, *n*_*c*_ is the number of channels of the feature map, *f* is the filter size, and *s* is the length of stride.

#### 4.1.3 Softmax layer

The softmax layer is the layer after the last convolution layer and is used to generate the probability distribution function for the output classification. The softmax function can be written as follows:

$$\sigma \;{\left(x\right)}_{i}=\frac{e^{xi}}{\Sigma_{j=1}^{k}e^{xj}}\;for\;\;i=1,2,\ldots ,K\;and\;x=x_{1},x_{2},\ldots ,x_{k}$$ (6)

where *x* is the input vector for softmax, *x*_*i*_ is the elements of the input vector, and *K* is the number of output classes.

The standard exponential function is applied on each *x*_*i*_ of the input vector *x*, and these values are normalized by dividing by the sum of all exponentials. The sum of all output vectors is equal to zero, i.e., Σ *σ* (*x*) = 0.

#### 4.1.4 Classification layer

This layer performs the classification of each image according to its probability distributions. The output size of the preceding layer is used to estimate the number of classes. To specify the three classes, we have to set a fully connected layer for output size to three and then a softmax layer is used. To use deep neural networks for classification tasks, the last fully connected layer of the CNN was replaced with an average pooling layer, followed by a fully connected layer that has three output nodes for three classes (normal, early, and advanced), two output nodes for two classes (normal and glaucoma), and a softmax classifier.

ResNet50 is a CNN that has 48 convolutional layers, along with one max pool and one average pool layer. This network input image size is 244 × 244. It learned from over a million images in the ImageNet collection. It has the ability to categorize images into 1,000 different object types. VGG19 is a CNN that has been trained on over a million images from the ImageNet dataset. There are 19 layers in total. VGG19 requires a 244 × 244 input image. It has sixteen convolutional layers, three fully connected layers, and a softmax layer at the end. AlexNet is made up of eight levels. It was trained on the ImageNet dataset, which has over 14 million images classified into 1,000 classes. It consists of five layers along with max-pooling layers followed by three fully connected layers, and they use the relu function in each of these layers other than the output layer, which also has two dropout layers. The input image size of VGG19 is 227 × 227. Dns201 is a CNN that is 201 layers deep. We can download a pre-trained version of the network from the ImageNet database that was trained on more than a million photos. It can categorize photos into 1,000 different object categories. The network can accept images up to 224 × 224 pixels. A CNN named InsRes was trained using more than a million photos from the ImageNet collection. The network has 164 layers and can categorize images into 1,000 object types, including keyboard, mouse, pencil, and numerous animals. The network has, therefore, acquired rich feature representations for a variety of images. The size of the network’s image input is 299 × 299 pixels.

#### 4.1.5 Data augmentation

**FIGURE 3:**
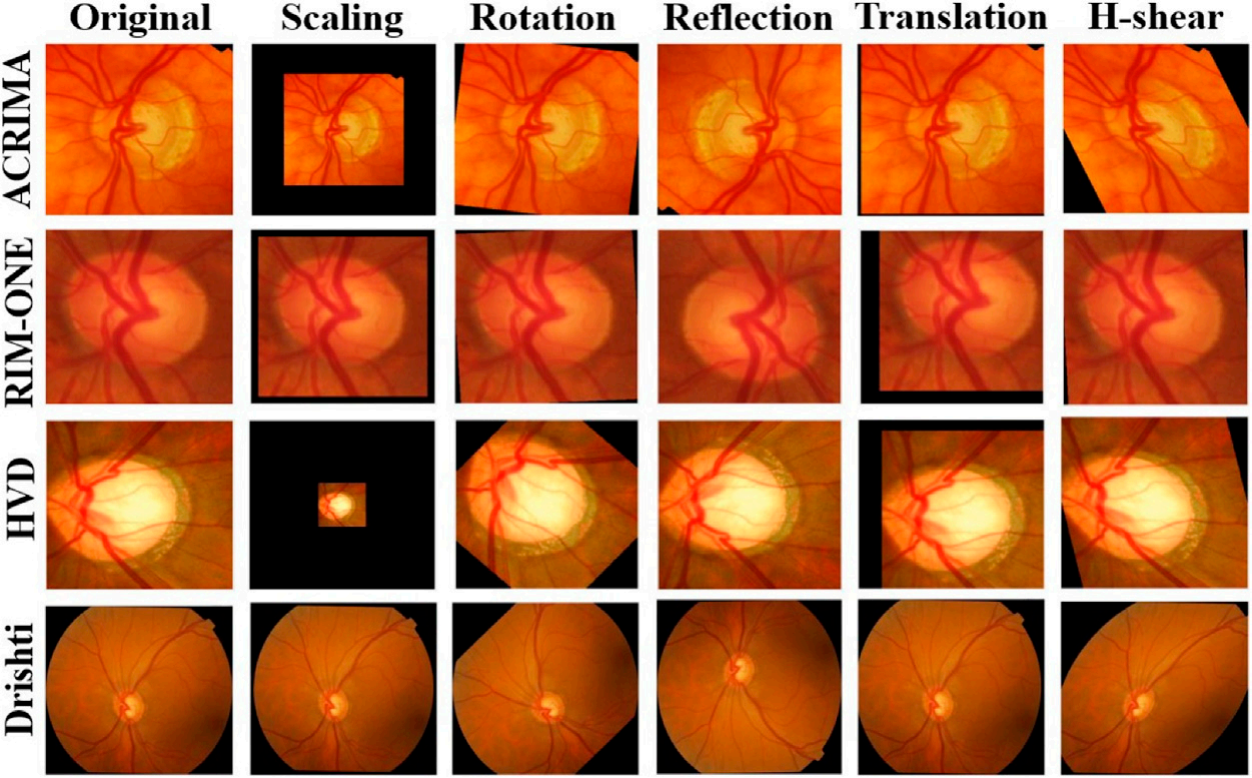
Comparison of augmented images of glaucoma class of each dataset.

Every dataset was resized to the default input size of each CNN model and also used data augmentation to increase the size of the dataset with augmentation options random scale, random translations and reflections about horizontal and vertical axes, random rotation to escape from overfitting, and boost the stability of the models. These augmentation options which we applied are shown in Table 2, and the comparison of augmented images corresponding to the glaucoma class of each dataset is shown in Figure 3.

**TABLE 2:** Data augmentation details.

Option	Value
RandScale	(0.05 and 1)
RandRotation	(−45 and 45)
RandXReflection	1
RandYReflection	1
RandXTranslation	(−50 and 50)
RandYTranslation	(−50 and 50)
RandXShear	(−30 and 30)

The purpose of all these options is to make the model more robust to changes in the orientation, position, or shape of the input images. The abovementioned options are commonly used because they are simple to implement, computationally efficient, and can help to increase the diversity of the training data without fundamentally changing the underlying content of the images. There are other image data augmentation options like noise, brightness, contrast, and saturation, but we have avoided them because the images in the datasets are already well-lit and have high contrast, so it may not be necessary to apply brightness or contrast adjustments. Also, adding noise is not a good decision because it may make the model difficult to extract meaningful features from the image.

The training options used in the training process are discussed as follows. The optimizer of adaptive moment estimation (Adam) is used in our program, given the batch size of 64 for three-class datasets and 8 for two-class datasets. We have set the execution environment to “CPU” and a learning rate of 1e-5 (0.00001). We have run the experiment with different epoch sizes, such as 20, 30, 40, and 50. Furthermore, we observed good performance when the epoch size is 50. The hyperparameters are adjusted to provide beneficial results in our tests. In addition to these tests, we measured the performance of the CNNs using the k-fold cross-validation approach with k = 5.

### 4.2 Classifier fusion

**FIGURE 4:**
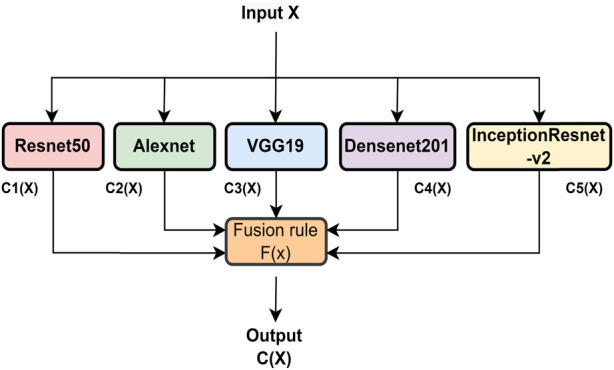
Classifier fusion to improve the performance of the system in glaucoma classification.

To enhance the classification performance of the system, a combination of three CNN models is employed, and their decisions are fused using a technique called classifier fusion (CF). The CF approach combines the outputs of multiple classifiers to arrive at a final decision. Different types of CFs exist, differing in their structure and type of fusion operation. In this study, the maximum voting-based fusion method is used. This involves subjecting the output of each CNN to a CF and taking the final decision based on the majority of votes. Majority voting is the most commonly used technique in voting-based decision-fusion methods. The CF operation is shown in Figure 4, where X is the input image and *C*1(*X*), *C*2(*X*), *C*3(*X*), *C*4(*X*), and *C*5(*X*) are the predicted output labels from ResNet50, AlexNet, VGG19, DenseNet, and Inception-ResNet, respectively, for the input image X. The fusion output *C*(*X*) can be expressed as follows:

$$C\;\left(X\right)=F\;\left(C1\;\left(X\right),C2\;\left(X\right),C3\;\left(X\right),C4\;\left(X\right),C5\;\left(X\right)\right)$$ (7)

where *F* (.) is the fusion rule. We have used the maximum voting-based rule for CF. For input “X,” the outputs of five CNN models are combined, and then, a maximum voting-based decision is considered among all. As a result, the “X” output class was determined.

Summarized classifier fusion results and summary of classification details of each class using CNN models of two-class dataset ACRIMA and three-class dataset HVD are shown in Table 3.

**TABLE 3:** Summarized glaucoma classification results of ACRIMA and HVD datasets by classifier fusion.

ACRIMA						
	ResNet50	VGG19	AlexNet	Dns201	IncRes	Fusion
Normal	N (309)	N (307)	N (302)	N (308)	N (309)	N (309)
(309)	G (0)	G (2)	G (7)	G (1)	G (0)	G (0)
Glaucoma	N (1)	N (8)	N (31)	N (6)	N (50)	N (3)
(396)	G (395)	G (388)	G (365)	G (390)	G (346)	G (393)
HVD						
Normal (788)	N (715)	N (682)	N (674)	N (670)	N (728)	N (710)
	E (48)	E (70)	E (75)	E (74)	E (34)	E (48)
	A (25)	A (38)	A (39)	A (44)	A (26)	A (30)
Early (289)	N (75)	N (61)	N (73)	N (60)	N (141)	N (71)
	E (155)	E (167)	E (137)	E (183)	E (104)	E (166)
	A (59)	A (61)	A (79)	A (46)	A (44)	A (52)
Advanced (467)	N (26)	N (20)	N (16)	N (21)	N (81)	N (16)
	E (24)	E (29)	E (21)	E (37)	E (54)	E (8)
	A (417)	A (418)	A (430)	A (409)	A (332)	A (443)

## 5 Results

The proposed method is tested in MATLAB R2022a on a Windows 10Pro system with an Intel (R) Xeon (R) W-2125 CPU @4.00 GHz 4.01 GHz processor and 32 GB RAM.

For all CNN models with different epoch sizes, five-fold cross-validation was used. It enables us to make better use of our data and gives us a lot more information about how well our CNNs are performing. Classification performance can be measured in different ways. The following are the most widely used performance measures for classification problems: confusion matrix, accuracy, sensitivity, specificity, precision, recall, f-measure, and gmean. The confusion matrix shows the predicted class and the actual class values in a table structure that allows visualization of the deep learning (Kim, 2018) model’s performance metric for binary or multi-class classification.

When comparing the system’s efficiency, the accuracy calculation is utilized. It considers the classifier’s total number of true predictions. The accuracy is evaluated by the following equation:

$$Accuracy\left(ACC\right)=\frac{TP+TN}{TP+TN+FP+FN}$$ (8)

Sensitivity (recall or the true positive rate) is the ratio of number of true positive predictions and the total number of positive predictions Sensitivity is evaluated as follows:

$$Sensitivity\left(SN\right)=\frac{TP}{TP+FN}$$ (9)

Specificity (true negative rate) is the ratio of true negative predictions and the total number of negative predictions. It is given as follows:

$$Specificity\left(SP\right)=\frac{TN}{TN+FP}$$ (10)

Precision is the number of positive class predictions that really belong to the positive class. The following equation can be used to calculate it:

$$\mathrm{Pr}ecision\left(PRE\right)=\frac{TP}{TP+FP}$$ (11)

F-measure generates a single score that combines precision and recall issues into a single value.

$$F-measure\left(FM\right)=\frac{2\left(\mathrm{Pr}ecision\right)\left(Recall\right)}{\left(\mathrm{Pr}ecison+Recall\right)}$$ (12)

The geometric mean (Gmean) is a metric that measures the balance between classification performances on both the majority and minority classes. A low Gmean is an indication of poor performance in the classification of the positive cases, even if the negative cases are correctly classified as such.

$$Gmean\left(GM\right)=\sqrt[{\;}]{SN\;*\;SP}$$ (13)

where *SN* is the sensitivity and *SP* is the specificity.

In all the aforementioned equations, true positive (TP) is the number of positively predicted labels when actual labels are positive, true negative (TN) is the number of negatively predicted labels when actual labels are negative, false positive (FP) is the number of positively predicted labels when actual labels are negative, and false negative (FN) is the number of negatively predicted labels when actual labels are positive. Using the equations introduced previously, the performance of each CNN model is evaluated and tabulated in Tables 4–8, respectively.

**TABLE 4:** Performance metric of the HVD dataset using different CNN models.

CNN model	AUC	ACC	SEN	SP	PRE	FM	GM
ResNet50	0.9703	0.8335	0.5363	0.9019	0.5467	0.5575	0.6955
VGG19	0.9680	0.8206	0.5778	0.8764	0.5466	0.5186	0.7116
AlexNet	0.9600	0.8038	0.8553	0.8797	0.4749	0.4757	0.6458
Dns201	0.9642	0.8174	0.8758	0.7920	0.6461	0.7436	0.8329
IncRes	0.9324	0.7539	0.7109	0.7725	0.5754	0.6306	0.7411
Fusion	—	**0.8543**	**0.8080**	**0.9214**	**0.8275**	**0.8129**	**0.8628**

Bold values are showing the model results after applying classifier fusion operation.

The performance of the HVD dataset is shown in Table 4. The ResNet50 dataset has given good accuracy when compared to the remaining models. Similarly, Tables 5–8 show the performance of the Drishti, HVD + Drishti, ACRIMA, and RIMONE dataset, respectively. The accuracies of three-class datasets HVD, Drishti, and HVD + Drishti by using ResNet50 are 83.35, 88.98, and 82.88%, respectively. Similarly, the accuracies of two-class datasets ACRIMA and RIMONE by ResNet50 are 99.86 and 92.53%, respectively. Then, we applied CF, which combines the results of all the CNNs, and that gives the output based on the maximum voting rule. For this fusion task, we have taken predicted labels of every CNN model and applied them to the CF and then made new predicted labels from the CF output. Then, we calculated the performance metric using these predicted labels and actual labels. We observed that there was a significant increment in the accuracy of all datasets when we applied a CF with accuracies of ACRIMA, Drishti, HVD + Drishti, HVD, and RIMONE datasets after taking fusion 99.57, 90.55, 85.18, 85.43, and 94.95%, respectively. The experiment has been performed in several trials with different numbers of fine-tuned training options and epochs to acquire satisfactory results for each model. Then, we inspected the effect of the number of epochs in which each design performed the best. We have performed the training of each model for different epoch sizes such that 20, 30, 40, and 50. The corresponding accuracies of all trials for the ACRIMA dataset are shown in Table 9. The proposed work for epoch size 50 has given the highest accuracy. The number of fine-tuned layers and epochs were adjusted, while other hyperparameters, such as optimizer, batch size, learning rate, and execution environment, were maintained constant.

**TABLE 5:** Performance metric of the Drishti dataset using different CNN models.

CNN model	AUC	ACC	SEN	SP	PRE	FM	GM
ResNet50	0.9436	0.8898	0.9398	0.7955	0.8966	0.9176	0.8646
VGG19	0.9339	0.8740	0.9639	0.7045	0.8602	0.9091	0.8241
AlexNet	0.8771	0.8425	0.9880	0.5682	0.8119	0.8913	0.7492
Dns201	0.9480	0.8661	0.9277	0.7500	0.8750	0.9006	0.8341
IncRes	0.9102	0.8346	0.9277	0.6591	0.8370	0.8800	0.7819
Fusion	—	**0.9055**	**0.7770**	**0.9259**	**0.8841**	**0.7770**	**0.8271**

Bold values are showing the model results after applying classifier fusion operation.

**TABLE 6:** Performance metric of the HVD + Drishti dataset using different CNN models.

CNN model	AUC	ACC	SEN	SP	PRE	FM	GM
ResNet50	0.9671	0.8288	0.8636	0.8118	0.6924	0.7686	0.8373
VGG19	0.9653	0.8306	0.8855	0.8037	0.6888	0.7749	0.8346
AlexNet	0.9479	0.7917	0.9455	0.7163	0.6205	0.7493	0.8230
Dns201	0.9671	0.8145	0.8400	0.8020	0.6754	0.7488	0.8208
IncRes	0.9318	0.7522	0.7600	0.7484	0.5971	0.6688	0.7542
Fusion	—	**0.8518**	**0.7992**	**0.9252**	**0.7969**	**0.7992**	**0.7980**

Bold values are showing the model results after applying classifier fusion operation.

**TABLE 7:** Performance metric of the ACRIMA dataset using different CNN models.

CNN model	AUC	ACC	SEN	SP	PRE	FM	GM
ResNet50	1.0000	0.9986	0.9975	1.0000	1.0000	0.9987	0.9987
VGG19	0.9989	0.9858	0.9798	0.9935	0.9949	0.9873	0.9866
AlexNet	0.9890	0.9461	0.9217	0.9773	0.9812	0.9505	0.9491
Dns201	0.9998	0.9901	0.9848	0.9968	0.9974	0.9911	0.9908
IncRes	0.9984	0.9291	0.8737	1.0000	1.0000	0.9326	0.9347
Fusion	—	**0.9957**	**0.9962**	**1.0000**	**0.9952**	**0.9962**	**0.9957**

Bold values are showing the model results after applying classifier fusion operation.

**TABLE 8:** Performance metric of the RIMONE dataset using different CNN models.

CNN model	AUC	ACC	SEN	SP	PRE	FM	GM
ResNet50	0.9721	0.9253	0.9200	0.9294	0.9109	0.9154	0.9247
VGG19	0.9330	0.8747	0.8900	0.8627	0.8357	0.8620	0.8763
AlexNet	0.9089	0.7604	0.4950	0.9686	0.9252	0.6450	0.6924
Dns201	0.9513	0.8945	0.8450	0.9333	0.9086	0.8756	0.8881
IncRes	0.9509	0.8989	0.8600	0.9294	0.9053	0.8821	0.8940
Fusion	—	**0.9495**	**0.9447**	**0.9843**	**0.9540**	**0.9447**	**0.9493**

Bold values are showing the model results after applying classifier fusion operation.

**TABLE 9:** Accuracies of each CNN model for the ACRIMA dataset for different epoch sizes.

CNN model	Accuracy (%)				
	Epoch = 20	Epoch = 30	Epoch = 40		Epoch = 50
ResNet50	97.58	97.73		98.01	**99.86**
VGG19	96.81	97.62		97.89	**98.58**
AlexNet	92.82	93.42		93.86	**94.61**
Dns201	97.53	97.81		98.34	**99.01**
InsRes	90.32	90.54		91.63	**92.91**

Bold values are showing the model results after applying classifier fusion operation.

**FIGURE 5:**
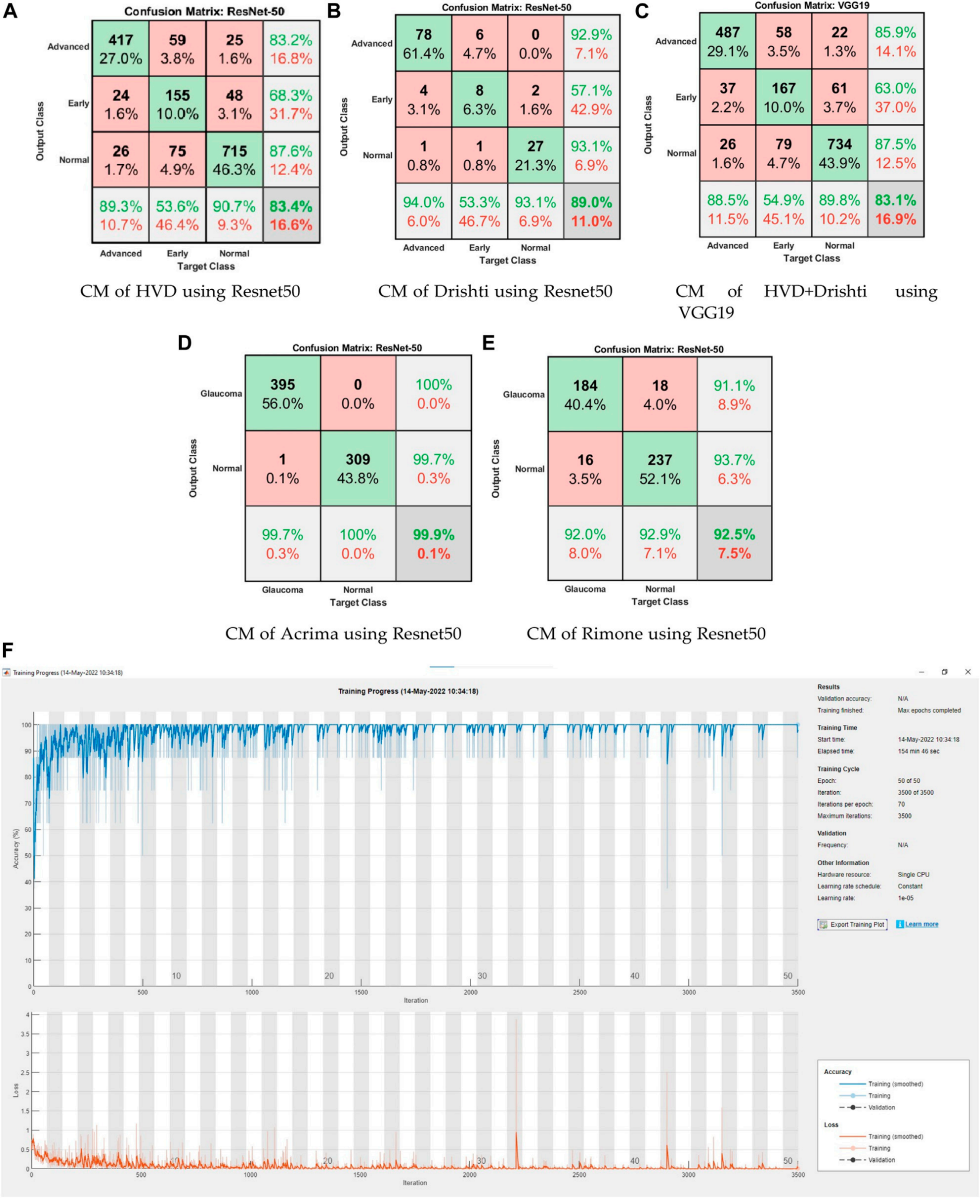
**(A–E)** Confusion matrices of two-class and three-class datasets using CNN models. **(F)** Training graph of ResNet50 for the ACRIMA dataset.

Time complexity of the training (training time) for individual network models, especially model training on the ACRIMA dataset, are provided in Table 10. An example training graph is shown in Figure 5F, where we can see the time elapsed for completing a fold. Generally, the time complexity of training a neural network depends on several factors, such as the number of layers, the number of neurons in each layer, the type of activation function used, and the size of the training data. Advancements in hardware and software technologies can significantly reduce the training time. For example, the use of graphics processing units (GPUs) for parallel processing can reduce the training time.

**TABLE 10:** Training time of CNN models on the ACRIMA dataset.

CNN model	Training time (in minutes)				
	Fold1	Fold2	Fold3	Fold4	Fold5
ResNet50	154.46	162.3	159.47	160.29	159.54
VGG19	338.20	343.36	343.48	350.13	334.48
AlexNet	69.52	69.41	69.5	70.11	70.19
Dns201	300.42	324.13	349.30	328.30	316.41
InsRes	472.22	509.16	496.26	488.47	518.59

**FIGURE 6:**
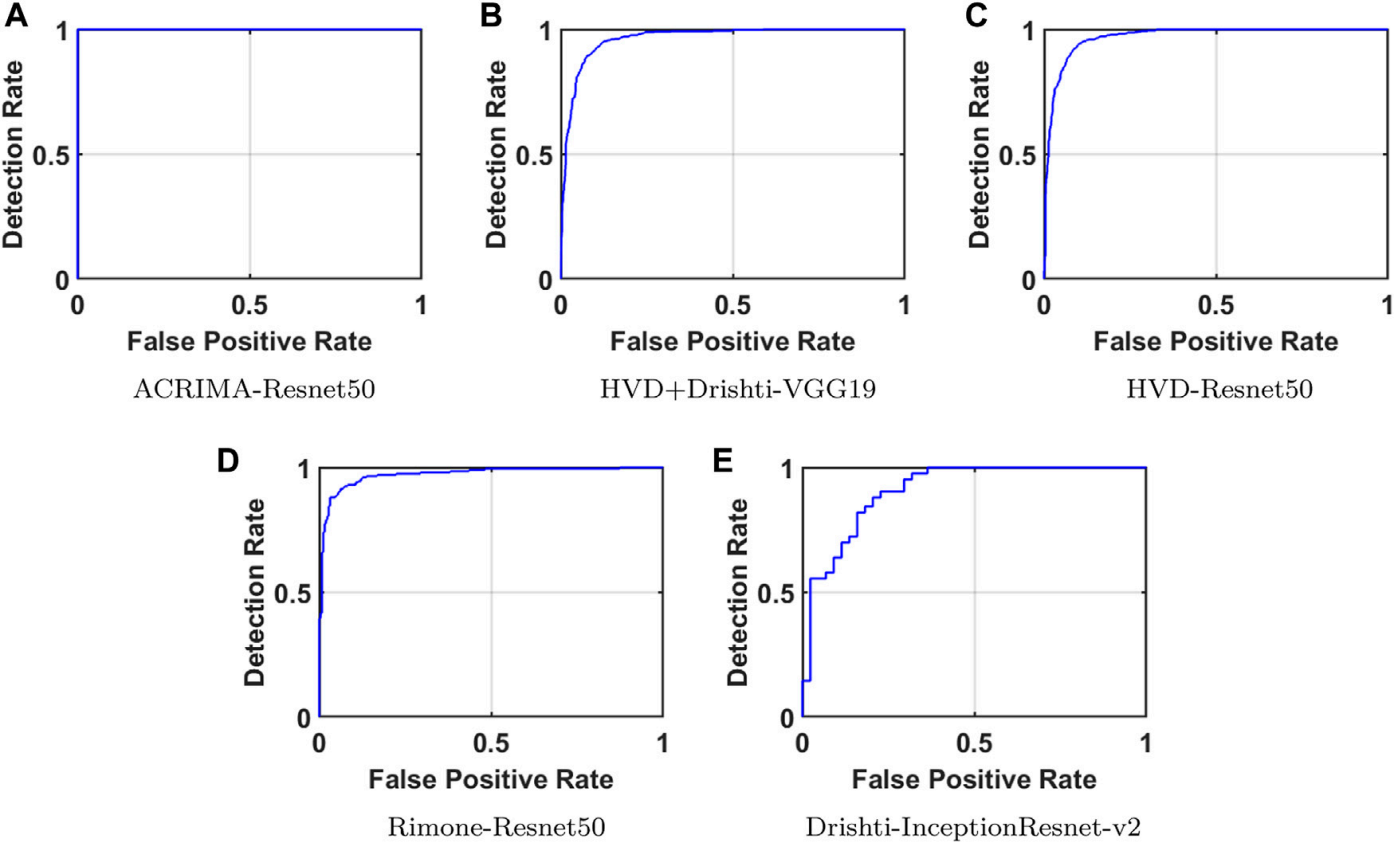
AUCs of CNN models for each dataset. **(A)** ACRIMA-ResNet50, **(B)** HVD+Drishti-VGG19, **(C)** HVD-ResNet50, **(D)** RIM-ONE-ResNet50, and **(E)** Drishti-Inception-ResNet-v2.

Confusion matrices (CMs) of each dataset for better accuracy among the five used CNN models are shown in Figures 5A–E. CMs of three-class dataset HVD, Drishti, and HVD + Drishti are shown in Figures 5A–C, respectively, and CMs of two-class dataset ACRIMA and RIMONE are shown in Figures 5D, E, respectively. The progress of the training graph of for k-fold cross-validation for k = 5, of ResNet50 for the ACRIMA dataset is shown in Figure 5F. All the training options which we used are appearing on the training graph as well. Time taken for completing one fold is also available in the graph, i.e., 154 min and 46 s. Figure 6 displays the AUCs obtained from the CNN models used in this study. An AUC value of 1 indicates that the classifier can accurately distinguish between all positive and negative class samples. Conversely, an AUC value of 0 would indicate that the classifier has misclassified all negative samples as positive and all positive samples as negative. For the ACRIMA dataset with ResNet50, we achieved an AUC of 1, which is shown in Figure 6A, AUC of the HVD + Drishti dataset for VGG19 with value 0.9318 is shown in Figure 6B, AUC of the HVD dataset for ResNet50 is 0.9324, shown in Figure 6C, AUC of the RIM-ONE dataset for ResNet50 is 0.9509, shown in Figure 6D, and an AUC of the Drishti dataset for Inception-ResNet with a value of 0.9102 is shown in Figure 6E.

## 6 Discussion

The discussion section includes the comparison of our results with the state-of-the-art methods and a sub-section to explain the network behavior with the help of deep learning visualization techniques. The subsequent paragraphs focus on comparing the outcomes of our proposed approach to those of the state-of-the-art techniques in glaucoma classification. Prior research on this topic mostly involved two-class glaucoma classification, whereas our contribution in this study is the development of an automatic glaucoma diagnostic system capable of three-class glaucoma classification. To assess the effectiveness of our approach, we compared our results to those of existing shallow learning-based state-of-the-art methods in Table 11 and compared our outcomes with deep learning-based state-of-the-art techniques in Table 12.

**TABLE 11:** Comparison with the existing machine learning-based state-of-art methods of glaucoma classification.

Author	Year	Feature extraction	Classifier	Database	No. of classes	Accuracy (%)
Li et al. (2023)	2023	ML models	SVM and RF	Private dataset	Two-class	79 for original data,
						84 for Compensated data
Khan et al. (2022)	2022	Wavelet-based	SVM	Private dataset	Two-class	91.22
		denoising and ML				
Shinde. (2021)	2021	U-Net and L-Net	SVM	RIM-ONE, Drishti-GS,	Two-class	99 for L-Net,
				DRIONS-DB, JSIEC, and DRIVE		98.67 for ROI
Huang et al. (2010)	2021	Entropy-based	LDA ANN	Private dataset	Two-class	NA,
						AUC:0.95 AUC:0.97
Noronha et al. (2014)	2020	HOS cumulant	SVM and NB	Private dataset	Three-class	92.65 Average accuracy
						84.75 for mild stage
Mohamed et al. (2019)	2019	Histogram	SVM	RIM-ONE	Two-class	98.6
		and texture				
Kishore and Ananthamoorthy. (2020)	2020	Intra-class and extra-class discriminative correlation analysis (IEDCA)	SVM, KNN and RF	HRF and DRIVE	Two-class	98.2 for HRF,
						97.7 for DRIVE
Proposed method	—	Pre-trained CNNs and classifier fusion	—	HVD	Three-class	85.43
				Drishti	Three-class,	**90.55**
				HVD + Drishti	Three-class	85.18
				ACRIMA	Two-class	**99.57**
				RIMONE	Two-class	94.95

Bold values are showing the model results after applying classifier fusion operation.

**TABLE 12:** Comparison with the existing deep learning-based state-of-the-art methods of glaucoma classification.

Author	Year	Method and classifier	Database	No. of classes	Accuracy (%)
Akbar et al. (2022)	2022	Transfer learning and fusion	HRF, RIM-1, and ACRIMA	Two-class	99.7, 89.3, and 99
Kumar and Gupta. (2022)	2022	Transfer learning models	Private data	Two-class	98.9
Rehman et al. (2021)	2021	Pre-trained deep CNN	ACRIMA, ORIGA, RIM-ONE,	Two-class	99.5
		architectures	AFIO, and HM		
Ajitha et al. (2021)	2021	Customized CNN	HRF, ORIGA, and Drishti-GS1	Two-class	93.86
Olivas et al. (2021)	2021	CNNs (MobileNet and	Collected from ZeissOCT machine	Two-class	90
		Inception V3)	at the Instituto de la Vision		
Li et al. (2019)	2020	Attention-based CNN	CGSA, Beijing Tongren and used	Two-class	96.2 for LAG,
			LAG and RIM-ONE for validation		85.2 for RIM-ONE
G´omez-Valverde et al. (2019)	2019	CNNs	ESPERANZA, Drishti and	Two-class	88.05
			RIM-ONE		
Diaz-Pinto et al. (2019b)	2019	Pre-trained CNNs	ACRIMA, HRF, Drishti, RIM-ONE,	Two-class	90.29
			and sjchoi86-HRF		
Proposed method	—	Pre-trained CNNs and classifier fusion	HVD	Three-class	85.43
			Drishti	Three-class,	**90.55**
			HVD + Drishti	Three-class	85.18
			ACRIMA	Two-class	**99.57**
			RIMONE	Two-class	94.95

Bold values are showing the model results after applying classifier fusion operation.

For the machine learning approaches, Li et al. (2023) proposed a model for glaucoma detection using ML models with a two-class-labeled private dataset, and achieved accuracies of 79 and 84% for original and compensated datasets, respectively. Khan et al. (2022) developed a glaucoma detection model using wavelet transform and ML model. They tested their model on the private dataset and achieved 91.22% accuracy. Kishore and Ananthamoorthy (2020) used k-nearest neighbors and random forest for feature extraction, which are machine learning supervised algorithms and support vector machine for the classification of glaucoma. They used two-class datasets, HRF and DRIVE, and received 98.2% accuracy for the HRF dataset and 97.7% accuracy for the DRIVE dataset. Shinde (2021) developed U-net and L-net methods and SVM classifier for glaucoma classification with two-class datasets which are RIM-ONE, Drishti-GS, DRIONS-DB, JSIEC, and DRIVE and received 99% accuracy for L-net. In the deep learning approaches, Rehman et al. (2021) used two-class datasets for their classification work such as ACRIMA, ORIGA, RIM-ONE, AFIO, and HM. They used the knowledge of pretrained deep CNN architectures and received 99.5% accuracy. Ajitha et al. (2021) proposed customized CNN architecture for the classification of glaucoma using two-class datasets HRF, ORIGA, and Drishti-GS1. They achieved 93.86% accuracy. Olivas et al. (2021) used CNNs (MobileNet and Inception V3) for the classification task with a two-class dataset which is a private dataset collected from Zeiss OCT machine at the Instituto de la Vision and achieved 90% accuracy. Li et al. (2019) used attention-based CNN in their work, and they used three different datasets CGSA, the dataset collected from Beijing Tongren hospital and the LAG dataset and the RIM-ONE dataset used for validation, and achieved 96.2% accuracy for the LAG dataset and 85.2% for the RIM-ONE dataset. G´omez-Valverde et al. (2019) used two-class datasets ESPERANZA, DRISHTI- GS, and RIM-ONE for their work and deep CNNs for glaucoma classification and achieved 88.05% accuracy. Diaz-Pinto et al. (2019b) used pre-trained CNNs for classification of glaucoma and achieved90.29% accuracy. They used two-class datasets for their work such as ACRIMA, HRF, Drishti- GS1, RIM-ONE, and sjchoi86-HRF. When comparing with the abovementioned state-of-the-art methods, the proposed novel method, which is a combination of transfer learning by deep CNNs and a classifier fusion, achieved 99.57% accuracy for the two-class dataset and achieved 90.55% for the three-class dataset.

### 6.1 Visualization techniques for understanding the behavior of the network to predict the input image class

**FIGURE 7:**
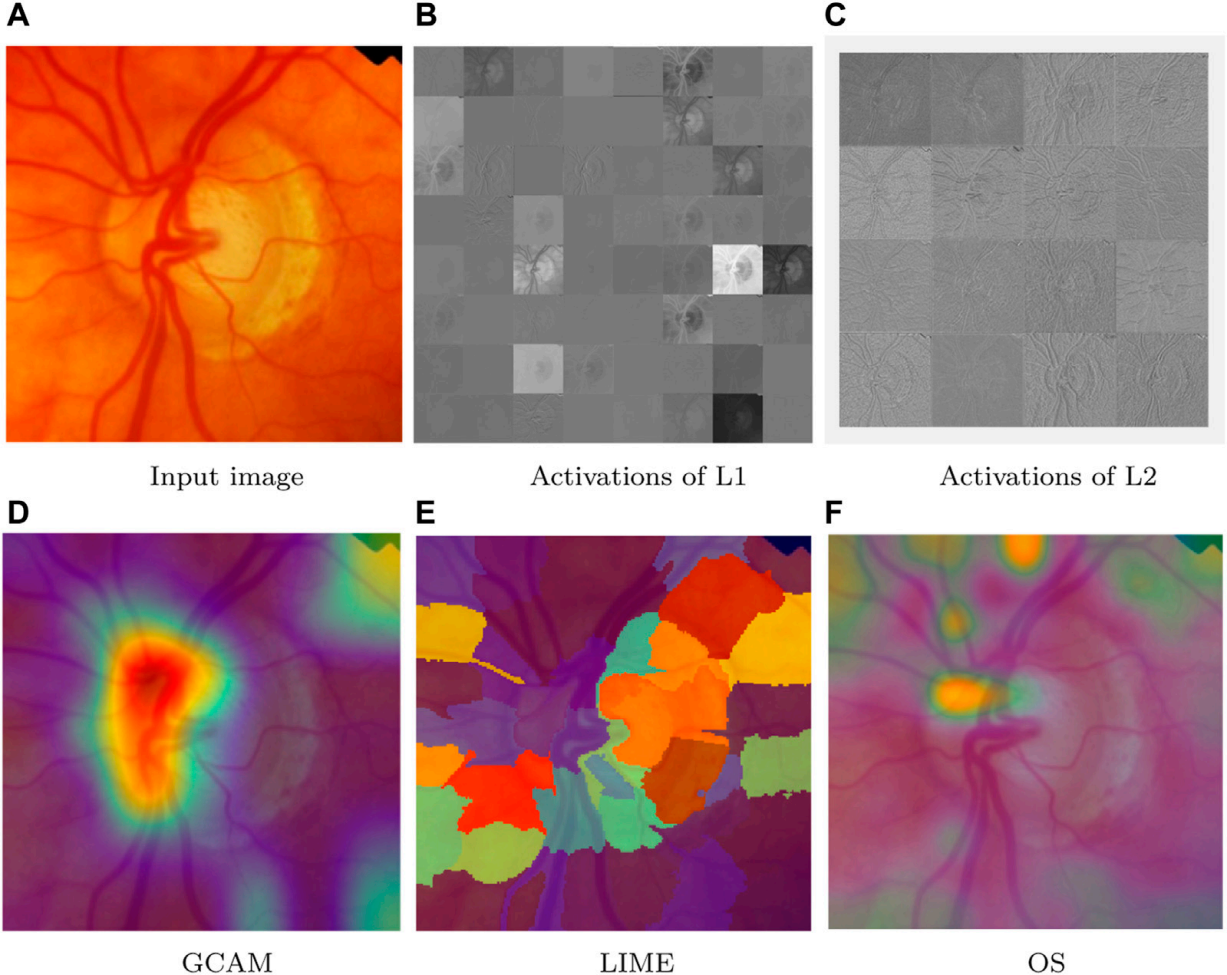
Network predictions for class detection using visualization techniques. **(A)** Input image, **(B)** activations of L1, **(C)** activations of L2, **(D)** GCAM, **(E)** LIME, and **(F)** OS.

Visualization techniques are used for investigating which area in the image is useful for the classification of class (Karaddi and Sharma, 2022). Every visualization technique has a unique methodology that influences the results it generates. The input to the network is perturbed through perturbation-based algorithms, which also take into account how the perturbation affects prediction. We have used input image from ACRIMA dataset for discussing the visualization techniques for network predictions by identifying the components more used for classification and it is shown in Figure 7A.

#### 6.1.1 Activations

A quick technique to comprehend the network behavior is to visualize activations. In the first convolutional layer, CNNs learn to recognize characteristics like color and edges. The activations of the first convolution layer (L1 = conv1) of ResNet50 are shown in Figure 7B, and it shows the edges which are present in the input image. Edge detection allows us to examine image features for significant changes in the grey level. The network learns to detect increasingly complex features as it moves through deeper convolutional layers. The activations of the deeper layer (L2 = res2c branch2a) are shown in Figure 7C.

#### 6.1.2 Gradient-based class activation heatmap

It is a heatmap of class activation depending on gradients. An extension of the CAM technique called gradient-weighted class activation mapping (Grad-CAM) makes use of the classification score’s gradient with regard to the convolutional features chosen by the network to decide which areas of the image are most crucial for classification. The final score is more strongly influenced by the data in the regions where the gradient is the largest. The Grad-CAM image corresponding to the input ACRIMA dataset is shown in Figure 7D. The area that is more helpful for class identification is shown by the red color. However, the yellow color region is involved moderately for class prediction, and blue color is the area which is not involved in the classification task.

#### 6.1.3 Locally interpretable model-agnostic explanations

LIME is a perturbation-based proxy model. It is a method for identifying the components of an image that a network uses when making a classification decision. The image LIME algorithm creates multiple synthetic images by randomly inserting or excluding features after segmenting an image into features. The excluded features are rendered useless for the network by having each pixel replaced with the average value of the image. The LIME image corresponding to the input ACRIMA dataset is shown in Figure 7E.

#### 6.1.4 Occlusion sensitivity

Occlusion sensitivity (OS) gauges how sensitive a network is even to slight changes in the input data. The technique alters small portions of the input by swapping out the original data for an occluding mask, usually a gray square. The method assesses the variation in the probability score for a specific class as the mask goes across the image. The most crucial areas of the image for classification can be highlighted using OS. OS visualization for the input image of the ACRIMA dataset is shown in Figure 7F. Visualization methods are a type of interpretability technique that uses visual representations of what a network is looking at to explain network predictions. Each visualization method has a distinct approach that determines the output. The methods can be local, examining network behavior for a single input, or global, examining network behavior over a dataset.

## 7 Conclusion

In conclusion, automated glaucoma diagnosis is crucial for the early detection and prevention of the disease. Traditional methods are time consuming, manual, and inaccurate. In this paper, a model for automated glaucoma stage classification is proposed, which uses five pre-trained deep CNN models and is tested with four public datasets. The proposed model achieved excellent results, with an AUC of 1 and an accuracy of 99.57% for the ACRIMA dataset, an AUC of 0.97 and an accuracy of 85.43% for the HVD dataset, and accuracy rates of 90.55% and 94.95% for Drishti and RIMONE, respectively. The proposed CF using the maximum voting-based approach (MVB) further improved the overall system performance. Furthermore, the activations, G-CAM, LIME, and OS approaches were used to generate class-specific heatmap images in order to show the area where the model is paying the most attention while making a decision. The experimental findings demonstrate that the proposed model outperforms the state-of-the-art methods in classifying glaucoma in its early stages, indicating its potential in improving the early detection and diagnosis of glaucoma, which can help prevent vision loss and permanent blindness. Finally, the proposed work could help ophthalmologists make a quick, accurate, and efficient glaucoma diagnosis.

Further research can be conducted to improve the proposed automated glaucoma diagnosis model, which would include potential areas like larger datasets that gives generalizability, integration of clinical data (patient age, sex, and medical history), and real-world implementation.

## Data Availability

Publicly available datasets were analyzed in this study. This data can be found at: ACRIMA- https://figshare.com/articles/dataset/CNNs_for_Automatic_Glaucoma_Assessment_using_Fundus_Images_An_Extensive_Validation/7613135, HVD- https://dataverse.harvard.edu/dataset.xhtml?persistentId=doi:10.7910/DVN/1YRRAC, RIMONE- https://medimrg.webs.ull.es/research/retinal-imaging/rim-one/, Drishti- http://cvit.iiit.ac.in/projects/mip/drishti-gs/mip-dataset2/Home.php.

## References

[B1] Acharya U. R. Dua S. Du X. Chua C. K. (2011). Auto-mated diagnosis of glaucoma using texture and higher order spectra features. IEEE Trans. informa- tion Technol. Biomed. 15 (3), 449–455. 10.1109/TITB.2011.2119322 21349793

[B2] Ajitha S. Akkara J. D. Judy M. (2021). Identification of glaucoma from fundus images using deep learning tech-niques. Indian J. Ophthalmol. 69 (10), 2702. 10.4103/ijo.ijo_92_21 34571619 PMC8597466

[B3] Akbar S. Hassan S. A. Shoukat A. Alyami J. Bahaj S. A. (2022). Detection of microscopic glaucoma through fun-dus images using deep transfer learning approach. Mi- croscopy Res. Tech. 85 (6), 2259–2276. 10.1002/jemt.24083 35170136

[B4] Alghamdi M. Abdel-Mottaleb M. (2021). A comparative study of deep learning models for diagnosing glaucoma from fundus images. IEEE access 9, 23894–23906. 10.1109/access.2021.3056641

[B5] Anwar S. M. Majid M. Qayyum A. Awais M. Al- nowami M. Khan M. K. (2018). Medical image analysis using con-volutional neural networks: A review. J. Med. Syst. 42 (11), 226–313. 10.1007/s10916-018-1088-1 30298337

[B6] Bock R. Meier J. Michelson G. Nyu´l L. G. Horneg- ger J. (2007). “Classifying glaucoma with image-based features from fundus photographs,” in Joint pattern recogni-tion symposium (Berlin, Germany: Springer), 355–364.

[B7] Chan Y. M. Ng E. Jahmunah V. Koh J. E. W. Lih O. S. Leon L. Y. W. (2019). Automated detec-tion of glaucoma using optical coherence tomography angiogram images. Comput. Biol. Med. 115, 103483. 10.1016/j.compbiomed.2019.103483 31698235

[B8] Cheng J. Liu J. Xu Y. Yin F. Wong D. W. K. Tan N.-M. (2013). Super-pixel classification based optic disc and optic cup seg-mentation for glaucoma screening. IEEE Trans. Med. imaging 32 (6), 1019–1032. 10.1109/TMI.2013.2247770 23434609

[B9] Christopher M. Belghith A. Bowd C. Proudfoot J. A. Goldbaum M. H. Weinreb R. N. (2018). Performance of deep learn-ing architectures and transfer learning for detecting glaucomatous optic neuropathy in fundus photographs. Sci. Rep. 8 (1), 16685–16713. 10.1038/s41598-018-35044-9 30420630 PMC6232132

[B10] De La Fuente-Arriaga J. A. Felipe-River´on E. M. Gardun˜o-Calder´on E. (2014). Application of vascular bundle displacement in the optic disc for glaucoma detec-tion using fundus images. Comput. Biol. Med. 47, 27–35. 10.1016/j.compbiomed.2014.01.005 24530536

[B11] Diaz-Pinto A. Colomer A. Naranjo V. Morales S. Xu Y. Frangi A. F. (2019). Retinal image synthesis and semi-supervised learning for glaucoma assessment. IEEE Trans. Med. imaging 38 (9), 2211–2218. 10.1109/TMI.2019.2903434 30843823

[B12] Diaz-Pinto A. Morales S. Naranjo V. K¨ohler T. Mossi J. M. Navea A. (2019). Cnns for automatic glaucoma assess-ment using fundus images: An extensive validation. Biomed. Eng. online 18 (1), 29–19. 10.1186/s12938-019-0649-y 30894178 PMC6425593

[B13] Fumero F. Alay´on S. Sanchez J. L. Sigut J. Gonzalez-Hernandez M. (2011). “Rim-one: An open retinal im-age database for optic nerve evaluation,” in 2011 24th international symposium on computer-based medical systems (CBMS) (New Jersey, United States: IEEE), 1–6.

[B14] G´omez-Valverde J. J. Ant´on A. Fatti G. Liefers B. Herranz A. Santos A. (2019). Automatic glaucoma classification using color fundus images based on convolutional neural networks and transfer learning. Biomed. Opt. express 10 (2), 892–913. 10.1364/BOE.10.000892 30800522 PMC6377910

[B15] Haleem M. S. Han L. Hemert J. v. Fleming A. Pasquale L. R. Silva P. S. (2016). Regional image features model for automatic classification be-tween normal and glaucoma in fundus and scanning laser ophthalmoscopy (SLO) images. J. med- ical Syst. 40 (6), 132–219. 10.1007/s10916-016-0482-9 PMC4834108 27086033

[B16] Haleem M. S. Han L. Hemert J. v. Li B. Fleming A. Pasquale L. R. (2018). A novel adaptive deformable model for automated optic disc and cup segmentation to aid glaucoma diagnosis. J. Med. Syst. 42 (1), 20–18. 10.1007/s10916-017-0859-4 PMC5719827 29218460

[B17] Hatanaka Y. Fukuta K. Muramatsu C. Sawada A. Hara T. Yamamoto T. (2009). “Automated measure-ment of cup-to-disc ratio for diagnosing glaucoma in retinal fundus images,” in World congress on medical physics and biomedical engineering (Munich, Germany: Springer), 198–200.

[B18] Huang M.-L. Chen H.-Y. Huang W.-C. Tsai Y.-Y. (2010). Linear discriminant analysis and artificial neural net-work for glaucoma diagnosis using scanning laser polarimetry–variable cornea compensation measure-ments in taiwan Chinese population. Graefe’s Archive Clin. Exp. Ophthalmol. 248 (3), 435–441. 10.1007/s00417-009-1259-3 20012983

[B19] Imran A. Li J. Pei Y. Akhtar F. Mahmood T. Zhang L. (2021). Fundus image-based cataract classification using a hybrid convolutional and recurrent neural net-work. Vis. Comput. 37 (8), 2407–2417. 10.1007/s00371-020-01994-3

[B20] Islam M. Roy A. Laskar R. H. (2018). Neural network based robust image watermarking technique in lwt domain. J. Intelligent Fuzzy Syst. 34 (3), 1691–1700. 10.3233/jifs-169462

[B21] Karaddi S. H. Sharma L. D. (2022). Automated multi-class classification of lung diseases from cxr-images using pre-trained convolutional neural networks. Expert Syst. Appl. 211, 118650. 10.1016/j.eswa.2022.118650

[B22] Kausu T. Gopi V. P. Wahid K. A. Doma W. Niwas S. I. (2018). Combination of clinical and multiresolution fea-tures for glaucoma detection and its classification us-ing fundus images. Biocybern. Biomed. En- gineering 38 (2), 329–341. 10.1016/j.bbe.2018.02.003

[B23] Khan S. I. Choubey S. B. Choubey A. Bhatt A. Naishadhkumar P. V. Basha M. M. (2022). Automated glaucoma detection from fundus images using wavelet-based de-noising and machine learning. Concurr. Eng. 30 (1), 103–115. 10.1177/1063293x211026620

[B24] Kim U. (2018). “Machine learn for glaucoma,” in Harvard Dataverse. 10.7910/DVN/1YRRAC

[B25] Kishore B. Ananthamoorthy N. (2020). Glaucoma classifica-tion based on intra-class and extra-class discriminative correlation and consensus ensemble classifier. Genomics 112 (5), 3089–3096. 10.1016/j.ygeno.2020.05.017 32470644

[B26] Krishnan M. M. R. Faust O. (2013). Automated glaucoma de-tection using hybrid feature extraction in retinal fundus images. J. Mech. Med. Biol. 13 (01), 1350011. 10.1142/s0219519413500115

[B27] Kumar Y. Gupta S. (2022). Deep transfer learning approaches to predict glaucoma, cataract, choroidal neovascular-ization, diabetic macular edema, drusen and healthy eyes: An experimental review. Archives Computa- tional Methods Eng. 30, 521–541. 10.1007/s11831-022-09807-7

[B28] Li L. Xu M. Liu H. Li Y. Wang X. Jiang L. (2019). A large-scale database and a CNN model for attention-based glaucoma detection. IEEE Trans. Med. imaging 39 (2), 413–424. 10.1109/TMI.2019.2927226 31283476

[B29] Li C. Chua J. Schwarzhans F. Husain R. Gi- rard M. J. Majithia S. (2023). Assessing the external validity of ma-chine learning-based detection of glaucoma. Sci. Rep. 13 (1), 558. 10.1038/s41598-023-27783-1 36631567 PMC9834286

[B30] Liao W. Zou B. Zhao R. Chen Y. He Z. Zhou M. (2019). Clinical interpretable deep learning model for glaucoma diagnosis. IEEE J. Biomed. health infor- matics 24 (5), 1405–1412. 10.1109/JBHI.2019.2949075 31647449

[B31] Liu S. Graham S. L. Schulz A. Kalloniatis M. Zangerl B. Cai W. (2018). A deep learning-based algorithm iden-tifies glaucomatous discs using monoscopic fundus pho-tographs. Ophthalmol. Glaucoma 1 (1), 15–22. 10.1016/j.ogla.2018.04.002 32672627

[B32] Mohamed N. A. Zulkifley M. A. Zaki W. M. D. W. Hussain A. (2019). An automated glaucoma screening system using cup-to-disc ratio via simple linear iterative clus-tering superpixel approach. Biomed. Signal Process- ing Control 53, 101454. 10.1016/j.bspc.2019.01.003

[B33] Mookiah M. R. K. Acharya U. R. Lim C. M. Pet- znick A. Suri J. S. (2012). Data mining technique for automated diagnosis of glaucoma using higher order spectra and wavelet energy features. Knowledge-Based Syst. 33, 73–82. 10.1016/j.knosys.2012.02.010

[B34] Noronha K. P. Acharya U. R. Nayak K. P. Martis R. J. Bhandary S. V. (2014). Automated classification of glaucoma stages using higher order cumulant features. Biomed. Signal Process. Control 10, 174–183. 10.1016/j.bspc.2013.11.006

[B35] Olivas L. G. Alf´erez G. H. Castillo J. (2021). Glaucoma detec-tion in latino population through OCT’s rnfl thickness map using transfer learning. Int. Ophthalmol- ogy 41 (11), 3727–3741. 10.1007/s10792-021-01931-w 34212255

[B36] Parashar D. Agrawal D. K. (2020). Automatic classification of glaucoma stages using two-dimensional tensor empirical wavelet transform. IEEE Signal Process. Lett. 28, 66–70. 10.1109/lsp.2020.3045638

[B37] Parashar D. Agrawal D. K. (2020). Automated classification of glaucoma stages using flexible analytic wavelet trans-form from retinal fundus images. IEEE Sensors J. 20 (21), 12885–12894. 10.1109/jsen.2020.3001972

[B38] Raghavendra U. Bhandary S. V. Gudigar A. Acharya U. R. (2018). Novel expert system for glaucoma identifica-tion using non-parametric spatial envelope energy spec-trum with fundus images. Biocybern. Biomed- ical Eng. 38 (1), 170–180. 10.1016/j.bbe.2017.11.002

[B39] Rahul J. Sharma L. D. (2022). Automatic cardiac arrhyth-mia classification based on hybrid 1-d cnn and bi-lstm model. Biocybern. Biomed. Engineer- ing 42 (1), 312–324. 10.1016/j.bbe.2022.02.006

[B40] Raja J. Shanmugam P. Pitchai R. (2021). An automated early detection of glaucoma using support vector ma-chine based visual geometry group 19 (VGG-19) con-volutional neural network. Wirel. Personal. Commu- nications 118 (1), 523–534. 10.1007/s11277-020-08029-z

[B41] Rehman A. U. Taj I. A. Sajid M. Karimov K. S. (2021). An ensemble framework based on deep cnns architec-ture for glaucoma classification using fundus photogra-phy. Math. Biosci. Eng. MBE 18 (5), 5321–5346. 10.3934/mbe.2021270 34517490

[B42] Roy A. Singha J. Devi S. S. Laskar R. H. (2016). Impulse noise removal using SVM classification based fuzzy filter from gray scale images. Signal Process. 128, 262–273. 10.1016/j.sigpro.2016.04.007

[B43] Serener A. Serte S. (2019). “Transfer learning for early and advanced glaucoma detection with convolutional neu-ral networks,” in 2019 Medical technologies congress (TIPTEKNO) (New Jersey, United States: IEEE), 1–4.

[B44] Serte S. Serener A. (2019). “A generalized deep learning model for glaucoma detection,” in 2019 3rd International symposium on multidisciplinary studies and innovative technologies (ISMSIT) (New Jersey, United States: IEEE), 1–5.

[B45] Shabbir A. Rasheed A. Shehraz H. Saleem A. Za- far B. Sajid M. (2021). Detection of glaucoma using retinal fundus images: A comprehen-sive review. Math. Biosci. Eng. 18 (3), 2033–2076. 10.3934/mbe.2021106 33892536

[B46] Sharma L. D. Sunkaria R. K. (2018). Inferior myocardial in-farction detection using stationary wavelet transform and machine learning approach. Signal, Image Video Process. 12 (2), 199–206. 10.1007/s11760-017-1146-z

[B47] Sharma L. D. Chhabra H. Chauhan U. Saraswat R. K. Sunkaria R. K. (2021). Mental arithmetic task load recognition using EEG signal and bayesian optimized k-nearest neighbor. Int. J. Inf. Technol. 13 (6), 2363–2369. 10.1007/s41870-021-00807-7

[B48] Shinde R. (2021). Glaucoma detection in retinal fundus im-ages using u-net and supervised machine learning algo-rithms. Intelligence-Based Med. 5, 100038. 10.1016/j.ibmed.2021.100038

[B49] Shorten C. Khoshgoftaar T. M. Furht B. (2021). Deep learn-ing applications for Covid-19. J. big Data 8 (1), 18–54. 10.1186/s40537-020-00392-9 33457181 PMC7797891

[B50] Sivaswamy J. Krishnadas S. R. Datt Joshi G. Jain M. Syed Tabish A. U. (2014). “Drishti-gs: Retinal image dataset for optic nerve head(onh) segmentation,” in Proceedigs of the 2014 IEEE 11th International Symposium on Biomedical Imag- ing (ISBI), Beijing, China, 29 April 2014 - 02 May 2014, 53–56. 10.1109/ISBI.2014.6867807

[B51] Soltani A. Battikh T. Jabri I. Lakhoua N. (2018). A new expert system based on fuzzy logic and image process-ing algorithms for early glaucoma diagnosis. Biomed. Signal Process. Control 40, 366–377. 10.1016/j.bspc.2017.10.009

[B52] Sommer A. Tielsch J. M. Katz J. Quigley H. A. Gottsch J. D. Javitt J. (1991). Relationship between in-traocular pressure and primary open angle glaucoma among white and black americans: The baltimore eye survey. Archives Ophthalmol. 109 (8), 1090–1095. 10.1001/archopht.1991.01080080050026 1867550

[B53] Sreng S. Maneerat N. Hamamoto K. Win K. Y. (2020). Deep learning for optic disc segmentation and glaucoma diag-nosis on retinal images. Appl. Sci. 10 (14), 4916. 10.3390/app10144916

[B54] Sułot D. Alonso-Caneiro D. Ksieniewicz P. Krzyzanowska-Berkowska P. Iskander D. R. (2021). Glau-coma classification based on scanning laser ophthalmo-scopic images using a deep learning ensemble method. Plos one 16 (6), e0252339. 10.1371/journal.pone.0252339 34086716 PMC8177489

[B55] Tham Y.-C. Li X. Wong T. Y. Quigley H. A. Aung T. Cheng C.-Y. (2014). Global prevalence of glaucoma and projections of glaucoma burden through 2040: A systematic review and meta-analysis. Ophthalmology 121 (11), 2081–2090. 10.1016/j.ophtha.2014.05.013 24974815

[B56] Tulsani A. Kumar P. Pathan S. (2021). Automated segmen-tation of optic disc and optic cup for glaucoma assess-ment using improved unet++ architecture. Biocyber- netics Biomed. Eng. 41 (2), 819–832. 10.1016/j.bbe.2021.05.011

[B57] Zulfira F. Z. Suyanto S. Septiarini A. (2021). Segmentation technique and dynamic ensemble selection to enhance glaucoma severity detection. Comput. Biol. Med. 139, 104951. 10.1016/j.compbiomed.2021.104951 34678479

